# Real-World Long-Term Safety of Statin–Fibrate Combination Therapy in Older Patients (≥75 Years): A European Cohort Analysis

**DOI:** 10.3390/geriatrics11050135

**Published:** 2026-09-16

**Authors:** Nikolaos Papadopoulos, John Doupis, Theodoros Angelopoulos, Elias Tsougos, Manuel Suarez Tembra, José Luis Diaz, Diego Bellido Guerrero, Maria Antonia Sanchez-Calavera, Juan Carlos Garcia Alvarez, Antonio Robles Iniesta, Sonia Redondo, Antonio Ruiz Garcia, Jose Antonio Diaz, Daniel Zambon Rados, Pere Alvarez Garcia, Daiana Ibarretxe, Belen Fraille, Jose Maria Tirado Moliner, Luis Enrique Morales Cobo, Maria Jesus Barreda, Juan Luis Frigola, Fernando Gomez Peralta, Juan Carlos Moreno, Nuria Font, Antonio Fernandez, Jorge Manuel Romero Requena, José Porta, Stephanie Da Silva, Sophie De Niet

**Affiliations:** 1 Cardiology Clinic, AHEPA University General Hospital of Thessaloniki, Aristotle University of Thessaloniki, 54636 Thessaloniki, Greece; drnickpapadop@hotmail.com; 2 Diabetes Clinic and Clinical Research Center, Iatriko Paleou Falirou Medical Center, 17562 Athens, Greece; john.doupis@harvard-alumni.org; 3 Internal Medicine and Diabetes, Iatriko Paleou Falirou Medical Center, 17562 Athens, Greece; tangel28@yahoo.co.uk; 4 6th Cardiology Department, Hygeia Hospital, 15123 Athens, Greece; etsougos@gmail.com; 5 Lipids and Cardiovascular Risk Unit, San Rafael Hospital, 15006 A Coruna, Spain; msuareztembra@gmail.com; 6 Lipids and Cardiovascular Risk Unit, Hospital Abente y Lago, 15006 A Coruna, Spain; jose.luis.diaz.diaz@sergas.es; 7 Endocrinology and Nutrition, University Hospital of Ferrol, 15405 A Coruna, Spain; diegobellido@gmail.com; 8 Casablanca Health Centre, University of Zaragoza, 50009 Zaragoza, Spain; 9 Primary Care Management, Centro de Salud Universitario Dr. Mendiguchía Carriche, Servicio Madrileño de Salud, 28914 Leganes, Spain; jccombarro@hotmail.com; 10 Department of Internal Medicine, Hospital General de Requena, 46340 Requena, Spain; antonioroblesiniesta@yahoo.es; 11 Centro de Salud El Espinillo, 28041 Madrid, Spain; sonia.redondo.depedro@gmail.com; 12 Lipid and Cardiovascular Prevention Unit, Pinto University Health Centre, 28320 Pinto-Madrid, Spain; antoniodoctor@gmail.com; 13 Internal Medicine, Clinico Universitario, 15706 Santiago de Compostela, Spain; jose.antonio.diaz.peromingo@sergas.es; 14 Lipids and Artherosclerosis Unit Department of Internal Medicine, Hospital Clinic Barcelona, 08036 Barcelona, Spain; dzambon@clinic.cat; 15 Hayge Private Foundation, 08840 Barcelona, Spain; pereag@gmail.com; 16 Vascular Medicine and Metabolism Unit, Hospital Universitari Sant Joan de Reus, 43204 Tarragona, Spain; daianaig@hotmail.com; 17 Trinitat Health Centre, 46010 Valencia, Spain; fraile_mbe@gva.es; 18 Centro de Salud de Burriana, 12530 Castellon, Spain; jtiradomol@yahoo.es; 19 Centro de Salud Universitario Las Américas, Parla, 28983 Madrid, Spain; lmoralesgapm10@gmail.com (L.E.M.C.); jcarlos.moreno@salud.madrid.org (J.C.M.); 20 Centro de Salud Paulino Prieto, 33009 Oviedo, Spain; rebarcla@yahoo.es; 21 Cap Sant Pere de Reus, Institut Català de la Salut, 43202 Reus, Spain; jlfrigola@gmail.com; 22 Endocrinology and Nutrition Unit, Segovia General Hospital, 40002 Segovia, Spain; fgomezperalta@gmail.com; 23 Requena Hospital, 46340 Valencia, Spain; nuriafontlurbe@hotmail.es; 24 Alta Resolucion de Utrera Hospital, 41710 Sevilla, Spain; ajfr32@gmail.com; 25 Internal Medicine, Perpetuo Socorro Hospital, 06010 Badajoz, Spain; jmromerorequena@hotmail.com; 26 Sagasta Ruisenores Health Centre, 50006 Zaragoza, Spain; jporta@salud.aragon.es; 27 Laboratoires SMB SA, Clinical Department, 1080 Brussels, Belgium; sdsil@smb.be

**Keywords:** older patients, dyslipidemia, pravastatin 40 mg/fenofibrate 160 mg fixed dose combination, safety

## Abstract

**Background**: Given the increasing prevalence of dyslipidemia and multimorbidity in the older patient population, the lack of safety data on combined statin–fenofibrate therapy in this age group represents a critical gap. The post hoc subgroup analysis of the POSE study addresses this issue. **Materials and Methods**: The POSE study was a 3-year, real-world, observational, comparative, non-interventional study conducted in Europe. Patients with mixed dyslipidemia treated either by a fixed-dose combination of pravastatin 40 mg/fenofibrate 160 mg or a moderate-intensity statin monotherapy were enrolled. Safety outcomes included the occurrence of renal or urinary disorders, musculoskeletal or connective tissue disorders, hepatobiliary disorders, cholelithiasis, thromboembolic events, pancreatitis, worsening diabetes mellitus, elevated blood homocysteine levels, interstitial pneumopathy, phototoxicity, and fatal or non-fatal cardiovascular events. Laboratory parameters related to lipid control and renal, muscular, and hepatic functions were additionally evaluated. The post hoc analysis focused on a subgroup of patients aged 75 years and older. **Results**: A total of 458 patients were included in the analysis. The majority had hypertension (79.3%) and diabetes mellitus (55.0%). Over the 3-year study period, the incidence rate of composite safety events was numerically higher with the combination, though this did not reach statistical significance (RR = 2.18 [95% CI = 0.99–4.81]). Renal and urinary disorders were more frequent with the combination (5.5% vs. 0.8%), whereas musculoskeletal disorders, aggravated diabetes mellitus, and cardiovascular events showed no significant difference between groups, as the 95% confidence interval included 1. Globally, there were no significant differences regarding evolution of the hepatic, muscle, and renal biological parameters. From baseline to Year 3, TG levels decreased by 44.3% with pravastatin/fenofibrate and by 10.7% with statin monotherapy, while HDL-C levels increased by 26.5% and 0.4%, respectively. **Conclusions**: This real-life observational post hoc subgroup analysis suggests that in patients aged 75 years and older with mixed dyslipidemia at high or very high CV risk, pravastatin 40 mg/fenofibrate 160 mg demonstrates an acceptable long-term safety profile. The combination offers additional TG lowering and HDL-C improvement beyond moderate-intensity statin monotherapy, without a significant increase in overall safety events over three years.

## 1. Introduction

The global population aged 80 years and older will reach approximately half a billion in the coming years, and cardiovascular disease (CVD) remains the leading cause of mortality worldwide [1,2,3]. In Europe, demographic aging is particularly pronounced: Eurostat projections indicate that the population aged 75–84 years in the European Union will increase by 56% between 2019 and 2050, reflecting the rapid transformation of the region’s age structure [4]. Large-scale meta-analyses conducted by the Cholesterol Treatment Trialists’ (CTT) Collaboration have demonstrated the efficacy of statins in major vascular events irrespective of age. However, individuals aged over 75 years have been consistently underrepresented in controlled trials evaluating lipid-lowering therapies, and data specifically addressing frail older populations remain scarce [1,5]. This underrepresentation is largely attributable to the frequent exclusion of older patients from clinical trials due to multiple comorbidities and the widespread use of polymedications. As a result, uncertainty persists regarding both the magnitude of cardiovascular (CV) benefit and the safety profile of statin therapy in advanced age. Beyond their proven efficacy, the use of statins in older adults raises concerns related to potential adverse effects and an increased risk of drug–drug interactions, reflecting the complex medication regimens commonly prescribed to this population. In the context of aging and increased vulnerability to chronic conditions, particularly CVD, the optimal management of dyslipidemia in older individuals is of paramount importance. Older patients frequently present with multiple coexisting conditions, including hypertension, diabetes mellitus (DM), and chronic kidney disease, which complicate the therapeutic management of dyslipidemia. Moreover, age-related alterations in lipid metabolism, characterized by elevated low-density lipoprotein cholesterol (LDL-C) and triglyceride (TG) levels, along with reduced high-density lipoprotein cholesterol (HDL-C) result in a distinct lipid profile that warrants tailored clinical attention. In this sense, the combination of statin therapy with a fibrate is supported by a strong mechanistic rationale, particularly in patients with mixed dyslipidemia. Regarding the use of fibrates, fenofibrate was investigated in the FIELD trial. Herein, the use of fenofibrate demonstrated a significant reduction in overall CV complications among patients with type 2 DM. Although the treatment did not significantly reduce the primary outcome of major coronary events, it did demonstrate a reduction in non-fatal myocardial infarctions and a reduction in coronary revascularizations [6]. Subsequently, the ACCORD-Lipid trial assessed the addition of fenofibrate to simvastatin, showing no overall CV benefit, but suggesting a potential benefit in the predefined subgroup with atherogenic dyslipidemia, defined by elevated TG and low HDL-C levels [7]. Notably, older participants represented only a small proportion of both trials and concerns remain regarding renal and hepatic safety, particularly when fenofibrate is used in combination with statin therapy [8]. Given the increasing prevalence of dyslipidemia and multimorbidity in the older population, the lack of safety data on combined statin/fenofibrate therapy in this age group represents a critical gap. To address this issue, this study presents a post hoc subgroup analysis of the POSE study [9], a real-world observational comparative cohort trial comparing the combination of pravastatin and fenofibrate to moderate-intensity statin monotherapy, with a three-year follow-up, focusing on patients aged 75 years and older, to evaluate both efficacy and safety outcomes in this underrepresented population.

## 2. Materials and Methods

### 2.1. Study Design

This observational, real-world, multicenter, open-label, comparative cohort study was carried out in three European countries; Greece, Spain, and Portugal, between 2016 and 2022. Depending on the timing of patient enrollment, the study design included both retrospective and prospective components. Participating physicians were selected according to a predefined recruitment strategy designed to ensure broad representation of routine dyslipidemia care at the national level. Selection considered physician specialty (general practitioners, cardiologists, internists, and endocrinologists), practice setting (hospital-based or private practice), and participating country. The clinical trial was conducted in accordance with good pharmacovigilance and clinical practices. The study protocol received approval from the Pharmacovigilance Risk Assessment Committee, as well as from the relevant competent authorities and ethics committees in each of the participating countries. The study was registered in the HMA-EMA RWD catalogues (EUPAS 13661). The reporting of this observational cohort study followed the Strengthening the Reporting of Observational Studies in Epidemiology guidelines (Table S1).

### 2.2. Interventions

Patients were eligible if they had been prescribed, or were planning to be prescribed, either a fixed-dose combination of pravastatin 40 mg/fenofibrate 160 mg (Pravafenix^®^, Laboratoires SMB SA, Brussels, Belgium) or a moderate-intensity statin. Moderate-intensity statins were defined in accordance with the 2019 ESC/EAS guidelines [10] as therapies expected to reduce LDL-C levels by approximately 30% to 50% including: atorvastatin 10 mg, lovastatin 40 mg, pravastatin 40 mg, simvastatin 20–40 mg, fluvastatin 40–80 mg, and rosuvastatin 5–10 mg. Patients were enrolled consecutively after treatment had been prescribed according to routine medical practice in each participating country. Treatment allocation was not determined by the study protocol. To ensure a balanced representation of the two treatment groups at each study site, enrollment was managed so that the difference in the number of patients between the groups did not exceed two. Follow-up was conducted according to routine clinical practice over a period of three years. Laboratory tests were performed by local laboratories.

### 2.3. Primary Objectives

The primary objective was to document the incidence of safety endpoints associated with the fixed-dose combination of pravastatin 40 mg and fenofibrate 160 mg, as well as with moderate-intensity statin monotherapy. The composite safety endpoint was defined as the proportion of patients experiencing at least one of the following adverse events: renal or urinary disorders, musculoskeletal or connective tissue disorders, hepatobiliary disorders, cholelithiasis, thromboembolic events, pancreatitis, worsening of DM, increased blood homocysteine levels, interstitial pneumopathy, or phototoxic reactions.

### 2.4. Secondary Objectives

The secondary objective was to assess safety by evaluating the incidence of fatal and non-fatal CV events. Additionally, laboratory parameters related to dyslipidemia management, as well as renal, muscular, and hepatic function, were monitored.

### 2.5. Study Population

The present post hoc analysis focused on a subgroup of patients aged 75 years and older. Patients were eligible for enrollment if they were currently receiving or were expected to receive at the time of inclusion, either moderate-intensity statin monotherapy or a combination of pravastatin 40 mg and fenofibrate 160 mg, for up to 12 months prior to entering the study. Patients were excluded if they were involved in other clinical trials, were taking additional lipid-lowering therapies involving fibrates, or if their medical records were not accessible at the start of study treatment in terms of statins and fenofibrate contra-indications (i.e., exclusion of patients with impaired renal function or familial monogenic hypercholesterolemia).

### 2.6. Data Collection

In this real-life study, baseline clinical and safety data were obtained either retrospectively from patient medical records or directly at the time of inclusion using an electronic data capture questionnaire. Upon inclusion, eligibility was confirmed, and information on demographics, medical history, dyslipidemia, and CVD was collected. Cardiovascular risk was assessed using the Systematic Coronary Risk Evaluation (SCORE) chart, adapted from the 2019 ESC/EAS guidelines [10]. Following enrollment, data on clinical characteristics, dyslipidemia treatment, concomitant medications, laboratory parameters, and adverse events were collected prospectively during annual follow-up visits for up to three years or until the end of study treatment, whichever came first.

### 2.7. Statistics

The sample size was determined to evaluate the hazard ratio for the composite safety endpoints in real-world clinical practice over a 3-year follow-up. Based on existing literature, it was anticipated that 1–5% of patients would experience one of the safety endpoints [7,11,12,13,14]. The present post hoc analysis included all patients aged 75 years and older identified in the parent POSE study. Accordingly, the sample size was fixed by the number of eligible patients available in the parent cohort (*n* = 458), and no formal sample size or power calculation was performed for this subgroup analysis. The analyses were exploratory, and the limited number of events restricted the precision of the estimates and the ability to detect modest differences between treatment groups, particularly for rare safety outcomes. Incidence rates for safety and CV events were estimated along with their 95% confidence intervals (CIs). Absolute incidence rates were calculated based on the number of patients experiencing at least one event during follow-up and reported by treatment group. Statistical differences between treatment groups were assessed using relative risks (RRs) with corresponding 95% CIs. A lack of statistical significance was concluded when the 95% CI of the RR included 1. In addition, hazard ratios (HRs) for safety events were estimated using Cox proportional hazards regression to account for time-to-event data. Continuous variables were expressed as mean values with 95% CIs and standard deviations (SDs), while categorical variables were presented as counts and percentages. All analyses were performed on the full analysis set using SAS software version 9.3. In the parent POSE study, propensity score adjustment was used to reduce confounding related to the non-randomized treatment allocation, as previously described in the parent study publication [9]. Because the present work represents a post hoc analysis restricted to patients aged 75 years and older, no new subgroup specific propensity score model was developed. Baseline balance was therefore evaluated using standardized mean differences (SMDs), and the findings were interpreted taking into account the possibility of residual confounding. Analyses were performed using available data, and no imputation of missing values was performed. No sensitivity analyses were performed.

## 3. Results

**Figure 1:**
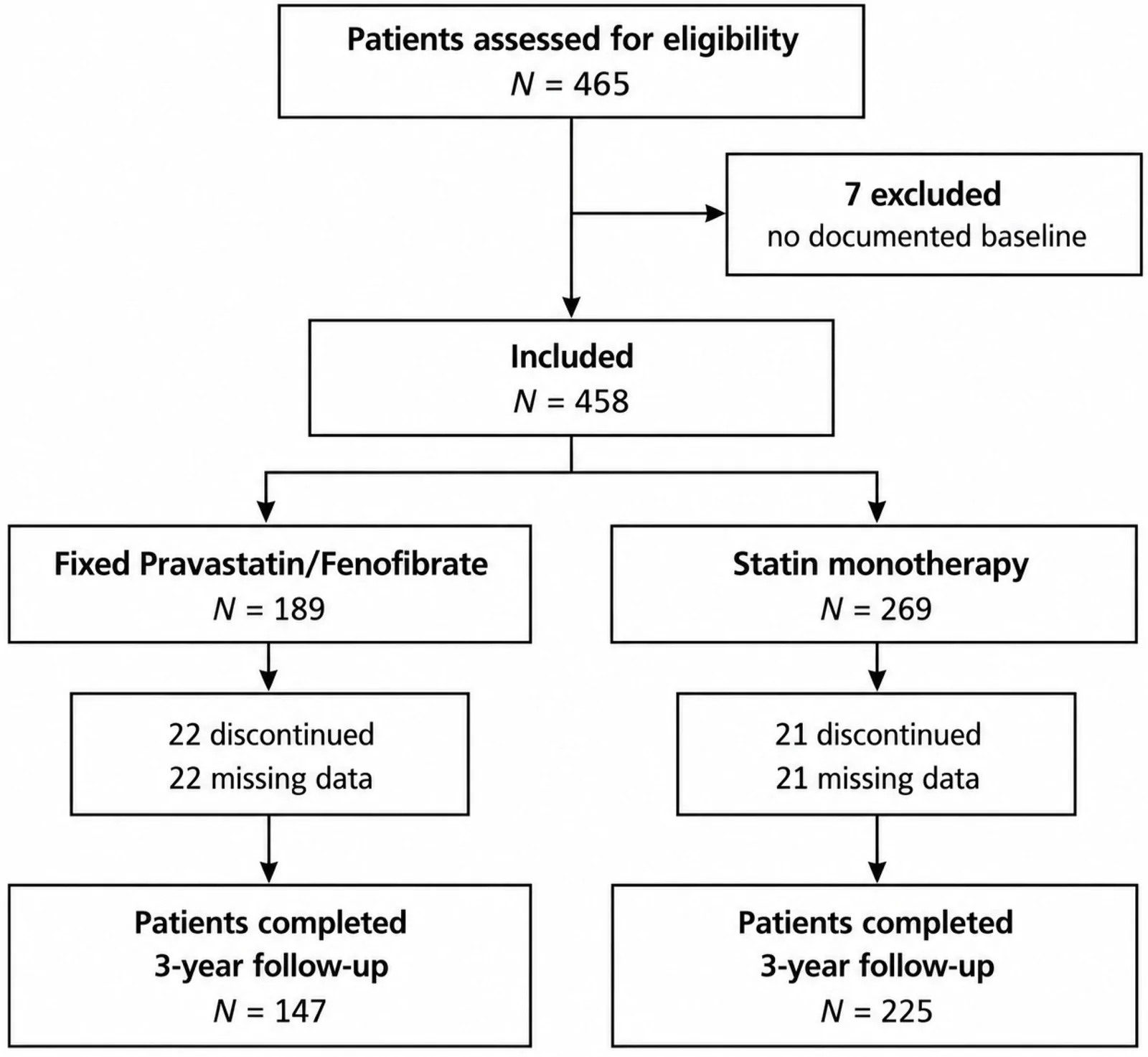
Study flowchart.

A total of 458 patients with a documented baseline visit and a signed informed consent form constituted the full safety analysis set, out of which 189 were treated with pravastatin 40 mg/fenofibrate 160 mg combination and 269 were treated with a moderate-intensity statin monotherapy. At the end of the 3-year follow-up period, 81.2% of the overall patients completed the study; 77.8% patients from the pravastatin 40 mg/fenofibrate 160 mg combination group, and 83.6% from the statin monotherapy group (Figure 1).

Treatment adherence was comparable between both treatment groups, with 11.1% of treatment change or discontinuation over the 3-year follow-up period in the fixed pravastatin 40 mg/fenofibrate 160 mg combination group and 8.2% in the statin group. Moderate imbalances were observed for sex, diabetes mellitus, and cardiovascular risk category, whereas most other baseline characteristics showed acceptable balance (Table 1). The mean age of the patients was 79.7 ± 3.8 years. More than half of the patients (57.4%) had a sedentary lifestyle and 7.2% were chronic smokers. The vast majority (79.3%) had hypertension and 55.0% had DM. Overall, 31.0% of patients had established CVD, reflecting a secondary prevention population, and 96.3% of the patients were classified as at high/very high CV risk level (97.9% in pravastatin 40 mg/fenofibrate 160 mg combination group and 95.1% in the statin group).

### 3.1. Incidence of Main Safety Events

Over the 3-year study period, the incidence rate of safety events was numerically higher with the combination, though this did not reach statistical significance (RR = 2.18 [95% CI = 0.99–4.81]). Notably, most events occurred during the first year of follow-up. In the fixed pravastatin 40 mg/fenofibrate 160 mg combination group, the absolute risk (AR) of the safety profile decreased from 4.9% at Year 1 follow-up to 3.7% at Year 2 and continued to fall to 1.3% by the Year 3 follow-up. Similarly, in the statin group, the AR of the safety profile decreased from 2.3% at the Year 1 follow-up to 2.0% at Year 2 and further declined to 0.9% by the Year 3 follow-up (Table 2). Across the 3-year follow-up, the composite safety incidence decreased in both treatment arms. The cumulative absolute risk was 9.3% with the fixed pravastatin 40 mg/fenofibrate 160 mg combination and 4.9% with statin monotherapy (RR 2.18; 95% CI 0.99–4.81). Notably, renal and urinary disorders were more frequent with the combination (5.5% vs. 0.8%; adjusted RR 6.47; 95% CI 1.42–29.48), whereas other safety categories, including musculoskeletal disorders, aggravated DM, and CV events, showed no significant between-group differences, as the 95% confidence interval included 1. Other safety events had either a very low global incidence (AR < 0.005 for thromboembolic events) or had a single event (cholelithiasis and hepatobiliary disorder) or even zero events (pancreatitis, blood homocysteine increase, interstitial pneumopathy and phototoxicity).

### 3.2. Incidence of CV Events

The global incidence of CV events collected was low (*n* = 8 events). The AR of fatal or non-fatal CV events over the cumulative three-year period was 0.022 (*n* = 4 events) in the pravastatin 40 mg/fenofibrate 160 mg combination group and 0.015 (*n* = 4 events) in the statin group. Over the 3-year study period, the RR for fatal and non-fatal CV events were 1.0, with a 95% confidence interval spanning 1 (0.36–5.67), indicating no significant differences between the two treatment arms, though interpretation is limited by the small number of events. In the fixed pravastatin 40 mg/fenofibrate 160 mg combination arm, the AR of fatal or non-fatal CV events declined from 0.011 (*n* = 2) at Year 1 to 0.000 (*n* = 0) at Year 3. In contrast, in the statin arm, the absolute risk increased from 0.004 (*n* = 1) at Year 1 to 0.009 (*n* = 2) at Year 3 (Table 2).

### 3.3. Lipids and Other Laboratory Parameters Outcome

The levels of total cholesterol, LDL-C, and TG decreased steadily in both groups from baseline to the 3-year follow-up (Table 3). In addition, the HDL-C levels increased in both groups. Specifically, in the combination group, the mean TG levels decreased from 2.7 ± 1.3 mmol/L at baseline to 1.5 ± 0.5 mmol/L after 3 years. In parallel, the mean HDL-C level increased from 1.2 ± 0.3 mmol/L at baseline to 1.3 ± 0.3 mmol/L after 3 years, whereas in the statin group, the mean TG level decreased from 1.5 ± 0.7 to 1.3 ± 0.4 mmol/L, and HDL-C remained stable at 1.3 ± 0.3 mmol/L. Laboratory safety parameters remained generally stable over the 3-year follow-up in both treatment groups. Changes in creatine phosphokinase, liver enzymes (AST and ALT), and creatinine clearance were of similar magnitude between groups among patients with available laboratory data. These laboratory findings should be interpreted separately from investigator-reported renal and urinary adverse events, which were analyzed as clinical safety outcomes (Table 4). Laboratory data were analyzed using all available observations. As expected in this real-world observational study, the number of available laboratory measurements decreased over time because laboratory assessments were performed according to routine clinical practice and were not mandatory at each follow-up visit. The number of available observations for each parameter is reported in Table 3 and Table 4. No imputation of missing values was performed.

## 4. Discussion

This real-life, observational post hoc analysis of the POSE study [9] suggests that in patients aged 75 years and older with mixed dyslipidemia and predominantly high or very high CV risk, long-term treatment with the fixed pravastatin 40 mg/fenofibrate 160 mg combination demonstrated an acceptable long-term safety profile over three years. From baseline to Year 3, the TG levels decreased by 44.3% with pravastatin/fenofibrate and by 10.7% with statin monotherapy, while the HDL-C levels increased by 26.5% and 0.4%, respectively. These findings should be interpreted descriptively, as baseline TG concentrations were higher in the combination group (2.7 vs. 1.5 mmol/L), suggesting that physicians may have preferentially prescribed the combination to patients with a more pronounced atherogenic/metabolic phenotype. This potential channeling bias may have influenced not only the observed lipid outcomes, but also the between-group differences in safety outcomes. Therefore, the observed differences cannot be attributed solely to treatment effects. The overall incidence of composite safety events remained relatively low in absolute terms in both groups. It was numerically higher with the combination, but this difference did not reach conventional statistical significance (adjusted RR = 2.18, 95% CI 0.99–4.81). Most safety events occurred during the first year and declined thereafter in both groups. Although these findings are consistent with acceptable long-term tolerability, they should be interpreted cautiously because the observed decline may also reflect treatment discontinuation, survivor bias (depletion of susceptible individuals), and incomplete follow-up inherent to a real-world observational study. Key safety considerations for fibrate/statin combination therapy primarily involve potential renal, hepatic, and muscle-related adverse events. This post hoc analysis, specifically focused on patients aged 75 years and older, is consistent with the data from the ACCORD-Lipid trial [7], with no new safety signals. The low overall incidence of safety events over three years was comparable with the incidence of moderate-intensity statin monotherapy. Over the 3-year follow-up, the musculoskeletal safety profile of pravastatin 40 mg/fenofibrate 160 mg was reassuring, with low rates of muscle-related events in both groups (2.2% vs. 1.5%) and no statistically significant differences between them. Importantly, no cases of rhabdomyolysis occurred in the combination group, despite the advanced age and frequent multimorbidity of the participants. Renal and urinary events, mainly related to creatinine elevations, were found to have low absolute event rates (5.5% vs. 0.8%), although were reported more often in the combination group over 3 years. Although renal and urinary disorders were reported more frequently in the pravastatin/fenofibrate group, this finding should be interpreted in the context of the different safety assessments performed in the study. Renal and urinary disorders represent investigator-reported clinical adverse events, whereas laboratory analyses describe longitudinal changes in renal function parameters in the subset of patients with available biological measurements. Consequently, these two analyses provide complementary rather than contradictory information. The higher incidence of renal adverse events was mainly driven by creatinine elevations, which are a recognized and generally reversible pharmacodynamic effect of fenofibrate and were not accompanied by evidence of progressive deterioration of renal function in the available laboratory dataset. Nevertheless, these were consistent with the established renal safety profile of fibrates, suggesting that these effects may be clinically manageable in older patients. Notably, in the ACCORD-Lipid trial [7] and consistent with the FIELD study [15], fenofibrate treatment slowed the progression of diabetic microvascular disease including retinopathy and nephropathy despite an early, sustained but reversible rise in serum creatinine. The PROSPER trial [16], which evaluated pravastatin 40 mg versus the placebo in nearly 20,000 adults aged 70–82 years, clearly demonstrated that statin therapy reduces the risk of coronary disease in older individuals, even over a relatively short 3-year period. However, the PROSPER study [16] evaluated only statin monotherapy, leaving unanswered the question of fenofibrate-pravastatin combination therapy in this age group. The ACCORD-Lipid [7] and FIELD [6] studies confirmed the benefit of adding fenofibrate to statin therapy. However, these trials were not designed to specifically evaluate older patients. In a large nationwide cohort study conducted by Ku et al. in 2024 [17], which included 114,920 patients treated with either fenofibrate plus a statin or statin monotherapy and followed for a median of 7.6 years, the authors found that among adults with type 2 DM who had been on statin therapy for more than one year, adding fenofibrate was associated with a reduced risk of peripheral vascular complications in older patients. Notably, the benefits of fenofibrate were more pronounced in older patients (age ≥ 65 years). The risk of acute kidney injury, rhabdomyolysis, or hospitalization for these events showed no significant difference between the two groups. In this post hoc analysis of the POSE study, the findings focused specifically on patients aged 75 years and older, a particularly fragile elderly population. In line with prior fibrate/statin trials conducted [PROSPER, ACCORD, FIELD, CTT], this study did not show a clear difference in the incidence of fatal or non-fatal CV events (2.2% for pravastatin/fenofibrate combination and 1.5% for statin monotherapy) between groups over three years. The absolute number of CV events was low (4 events in each group). However, the confidence intervals were wide, so the study was not powered to detect modest differences in this cohort. Nevertheless, the absence of a clear difference in CV events, combined with the observed lipid improvements provides supportive safety data for the use of a pravastatin/fenofibrate combination in appropriately selected older patients requiring additional TG lowering and HDL-C improvement beyond that achieved with moderate-intensity statin monotherapy.

A major strength of this post hoc sub-analysis is its specific focus on adults aged 75 years and older, an age group rarely represented in lipid-lowering trials but frequently affected by complex dyslipidemia, DM, and polypharmacy. Moreover, the high prevalence of hypertension, DM, and established CVD within the cohort reflects the complex clinical profile of older patients encountered in routine clinical practice. However, several limitations must be acknowledged. As this analysis was based on a subgroup of an observational cohort, residual confounding cannot be excluded. Although propensity score adjustment was performed in the parent study and baseline balance was assessed using standardized mean differences in the present subgroup, moderate differences remained for diabetes mellitus, sex, and cardiovascular risk category. Indeed, the pravastatin 40 mg/fenofibrate 160 mg combination group included a slightly higher proportion of women (62.4%) compared with the statin group (52.0%), a difference likely explained by the advanced mean age of the cohort (approximately 80 years) and the longer life expectancy of women [18]. There was a higher prevalence of two major CV risk factors in the pravastatin 40 mg/fenofibrate 160 mg combination group—arterial hypertension (82.0% vs. 77.3%) and DM (62.4% vs. 49.8%)—a pattern consistent with the mixed dyslipidemia phenotype and the age-related risk burden characteristic of this subpopulation. A further limitation is the potential for channeling bias. Baseline triglyceride levels were substantially higher in patients receiving pravastatin/fenofibrate than in those receiving statin monotherapy (2.7 vs. 1.5 mmol/L), suggesting preferential prescription of the combination to patients with a more pronounced atherogenic/metabolic phenotype. This underlying difference in patient profiles may have contributed not only to differences in lipid outcomes, but also to the observed differences in safety outcomes, including renal and urinary events. Consequently, these safety differences cannot be attributed solely to the treatment itself, which also limits their generalizability to older patients with different clinical and metabolic profiles. Another limitation is the relatively small number of cardiovascular and safety events limiting the statistical power to detect differences between treatment groups, particularly for rare outcomes. Consequently, the absence of statistically significant differences should not be interpreted as evidence of comparable safety, and the findings should be considered exploratory and interpreted with appropriate caution. An additional limitation concerns the geographical distribution and sampling strategy of the study population. Approximately 85% of participants in the parent POSE study were recruited in Greece, with substantially fewer patients enrolled in Spain and Portugal. Moreover, participating physicians were selected according to a predefined recruitment strategy rather than through probability sampling, and patients were consecutively enrolled in routine clinical practice. The study population should therefore be considered a predominantly Greek, non-probability sample. Consequently, the generalizability of the findings to the broader European population and other healthcare settings should be interpreted with caution.

## 5. Conclusions

This real-life observational post hoc subgroup analysis provides supportive long-term safety data on pravastatin 40 mg/fenofibrate 160 mg in patients aged 75 years and older with mixed dyslipidemia at high or very high CV risk. Although the cumulative incidence of composite safety events was numerically higher with the combination than with moderate-intensity statin monotherapy, the difference did not reach conventional statistical significance. Renal and urinary disorders were reported more frequently with the combination, although absolute event rates remained low, and no apparent deterioration in renal laboratory parameters was observed. These findings support the cautious use of the combination in appropriately selected older patients requiring additional TG lowering and HDL-C improvement, while reinforcing the importance of the routine monitoring of renal function during treatment. However, given the observational design, post hoc nature of the analysis, potential for residual confounding, non-probability sampling strategy, predominantly Greek study population, limited statistical power, and low number of events, these results should be considered supportive rather than confirmatory and should be extrapolated cautiously to other older patient populations.

## Figures and Tables

**Table 1:** Summary of the baseline characteristics.

Characteristic		Pravastatin 40/ Fenofibrate 160	Moderate-Intensity Statin	Standardized Mean Difference
		(*N* = 189)	(*N* = 269)	
Age (years)	Mean (SD)	79.76 (3.7)	79.71 (3.8)	0.013
Male	*n* (%)	71 (37.6)	129 (48.0)	0.211
Female	*n* (%)	118 (62.4)	140 (52.0)	0.211
Smokers	*n* (%)	11 (5.8)	22 (8.2)	0.094
Sedentary	*n* (%)	114 (60.3)	149 (55.4)	0.099
Weight (kg)	*n* (SD)	77.3 (12.14)	76.1 (12.37)	0.098
Waist circumference (cm)	*n* (SD)	97.3 (12.46)	96.7 (11.92)	0.049
Diabetes mellitus	*n* (%)	118 (62.4)	134 (49.8)	0.256
Very high CV risk	*n* (%)	72 (38.1)	123 (45.7)	0.154
High CV risk	*n* (%)	113 (59.8)	133 (49.4)	0.210
Moderate CV risk	*n* (%)	2 (1.1)	8 (3.0)	0.134
Low CV risk	*n* (%)	NA	NA	—
Hypertension	*n* (%)	155 (82.0)	208 (77.3)	0.117
Presence of established CVD	*n* (%)	52 (27.5)	90 (33.5)	0.131

*N*: number of patients; *n*: number of events; SD: standard deviation; CV: cardiovascular; NA: not applicable; CVD: cardiovascular disease.

**Table 2:** Absolute and relative risk of most frequent safety events at 1-year, 2-year, and 3-year follow-up.

		Absolute Risk ^a^		Relative Risk ^b^ (95% CI)
Variable	Timeline	Pravastatin/Fenofibrate (*N* = 183) ^d^	Statin (*N* = 263) ^d^	
Composite safety profile	Year 1	0.049	0.023	
	Year 2	0.037	0.020	
	Year 3	0.013	0.009	
	Over 3 years	0.093	0.049	2.178 (0.987; 4.810)
Musculoskeletal and connective tissue disorder	Year 1	0.005	0.008	
	Year 2	0.018	0.008	
	Year 3	0.000	0.000	
	Over 3 years	0.022	0.015	1.437 (0.364; 5.673)
Renal and urinary disorder	Year 1	0.027	0.004	
	Year 2	0.025	0.004	
	Year 3	0.007	0.000	
	Over 3 years	0.055	0.008	6.465 (1.418; 29.475)
Aggravated diabetes mellitus	Year 1	0.011	0.011	
	Year 2	0.006	0.008	
	Year 3	0.007	0.009	
	Over 3 years	0.022	0.027	1.024 (0.285; 3.688)
Thromboembolic events	Year 1	0.000	0.004	
	Year 2	0.006	0.000	
	Year 3	0.000	0.000	
	Over 3 years	0.005	0.004	NE ^c^
CV events	Year 1	0.011	0.004	
	Year 2	0.012	0.004	
	Year 3	0.000	0.009	
	Over 3 years	0.022	0.015	1 (0.364; 5.673)

CI: confidence interval; CV: cardiovascular; NE: not estimable. ^a^ Rate of new patients with at least one occurrence of a considered event. If a patient had experienced the same adverse event more than once, the patient was counted only for the first time he/she had experienced that adverse event. ^b^ Relative rate ratio evaluated by a log-binomial regression model with safety event as the response and quintiles of the propensity score and treatment group as predictors. Each safety event was a dummy variable equal to ‘1’ if the patient had experienced at least one occurrence during the follow-up period, otherwise the dummy variable was equal to ‘0’. Adjusted rate ratio for the composite safety profile, renal and urinary disorder, and thromboembolic events. Unadjusted rate ratio for musculoskeletal and connective tissue disorder and CV events. ^c^ Not estimable due to the low number of events. ^d^ Six patients in each treatment group had no evaluable follow-up safety data and were therefore not included in the incidence analyses.

**Table 3:** Summary of lipid laboratory parameters at baseline and 3-year follow-up.

Laboratory Parameter (Unit)	Visit		Pravastatin/Fenofibrate (*N* = 189)	Statin (*N* = 269)
Total cholesterol (mmol/L)	Baseline	*n*	185	265
		Mean (SD)	5.465 (1.256)	5.434 (1.257)
		Median	5.482	5.482
		Min, Max	3.31, 9.41	2.61, 8.25
	Year 3	*n*	98	164
		Mean (SD)	4.151 (0.632)	4.277 (0.881)
		Median	4.073	4.189
		Min, Max	2.51, 5.95	2.69, 7.76
	Change	%	−24.04	−21.29
HDL-C (mmol/L)	Baseline	*n*	179	259
		Mean (SD)	1.187 (0.282)	1.313 (0.480)
		Median	1.164	1.231
		Min, Max	0.59, 2.17	0.41, 5.09
	Year 3	*n*	94	157
		Mean (SD)	1.265 (0.273)	1.318 (0.299)
		Median	1.241	1.267
		Min, Max	0.65, 1.99	0.72, 2.20
	Change	%	26.5	0.38
LDL-C (mmol/L)	Baseline	*n*	177	259
		Mean (SD)	3.012 (1.039)	3.235 (1.108)
		Median	2.974	3.207
		Min, Max	0.64, 6.49	0.93, 6.26
	Year 3	*n*	96	161
		Mean (SD)	2.199 (0.596)	2.310 (0.720)
		Median	2.177	2.250
		Min, Max	0.96, 3.65	0.91, 5.07
	Change	%	−29.99	−28.59
Triglycerides (mmol/L)	Baseline	*n*	184	262
		Mean (SD)	2.702 (1.311)	1.513 (0.689)
		Median	2.703	1.409
		Min, Max	0.62, 8.07	0.13, 6.12
	Year 3	*n*	98	163
		Mean (SD)	1.504 (0.539)	1.351 (0.427)
		Median	1.403	1.288
		Min, Max	0.59, 3.74	0.47, 2.71
	Change	%	−44.34	−10.71

HDL-C: high-density lipoprotein cholesterol; LDL-C: low-density lipoprotein cholesterol; N: number of patients; *n*: number of events; SD: standard deviation.

**Table 4:** Summary of biological laboratory parameters at baseline and 3-year follow-up.

Laboratory Parameter (Unit)	Visit		Pravastatin/Fenofibrate (*N* = 189)	Statin (*N* = 269)
Creatine phosphokinase (ukat/L)	Baseline	*n*	118	151
		Mean (SD)	1.657 (1.357)	1.665 (1.256)
		Median	1.328	1.319
		Min, Max	0.28, 11.62	0.18, 9.34
	Year 3	*n*	57	80
		Mean (SD)	1.622 (0.846)	1.813 (1.030)
		Median	1.486	1.553
		Min, Max	0.45, 4.91	0.32, 5.63
	Change	%	−2.11	8.89
Aspartate aminotransferase (U/L)	Baseline	*n*	147	214
		Mean (SD)	19.766 (6.789)	21.409 (8.222)
		Median	19.000	20.000
		Min, Max	8.00, 42.00	7.00, 69.00
	Year 3	*n*	82	137
		Mean (SD)	21.896 (6.765)	22.227 (8.417)
		Median	21.500	21.000
		Min, Max	9.00, 39.00	9.00, 81.00
	Change	%	10.78	9.93
Alanine aminotransferase (U/L)	Baseline	*n*	150	216
		Mean (SD)	20.299 (11.763)	20.220 (12.279)
		Median	18.000	17.000
		Min, Max	6.00, 80.00	3.00, 90.00
	Year 3	*n*	84	137
		Mean (SD)	22.926 (10.394)	24.053 (14.394)
		Median	21.500	21.000
		Min, Max	7.00, 69.00	6.00, 95.00
	Change	%	12.94	18.96
Creatinine clearance (mL/s)	Baseline	*n*	44	46
		Mean (SD)	1.081 (0.320)	1.084 (0.386)
		Median	1.092	1.013
		Min, Max	0.38, 1.89	0.30, 2.04
	Year 3	*n*	24	35
		Mean (SD)	0.971 (0.277)	0.978 (0.249)
		Median	1.016	0.969
		Min, Max	0.22, 1.48	0.33, 1.54
	Change	%	−10.18	−9.78
Serum Creatinine (umol/L)	Baseline	*n*	165	223
		Mean (SD)	88.087 (23.501)	126.531 (592.702)
		Median	85.748	80.444
		Min, Max	45.97, 185.64	42.43, 8928.40 *
	Year 3	*n*	83	135
		Mean (SD)	95.712 (40.458)	89.625 (25.815)
		Median	89.284	85.748
		Min, Max	60.11, 420.78	44.20, 256.36
	Change	%	8.66	−29.17

*N*: number of patients; *n*: number of events; SD: standard deviation. * The value corresponds to a true outlier. Because laboratory parameters were analyzed descriptively and because the conclusions are based on the overall pattern of findings rather than on this single observation, the interpretation of the results remains unchanged.

## Data Availability

The datasets generated and analyzed during the current study contain pseudonymized clinical data from human participants. Public sharing is restricted by patient confidentiality requirements, the informed consent under which the data were collected, applicable data protection legislation (GDPR), and contractual obligations with the participating study centers. Data may be made available by the corresponding author upon reasonable request, provided that all applicable legal and ethical requirements are met.

## Supplementary Materials

The following supporting information can be downloaded at: https://www.mdpi.com/article/10.3390/geriatrics11050135/s1, Table S1: STROBE Statement—Checklist of items that should be included in reports of cohort studies.
